# Individuals' Awareness of and Willingness to Accept Hospital-at-Home Services and Related Factors: A Cross-Sectional Study

**DOI:** 10.3389/fpubh.2022.823384

**Published:** 2022-05-27

**Authors:** Siyu Xu, Jingjun Wang, Ya Wang, Mengmeng Wang, Xia Huang, Hao Huang

**Affiliations:** ^1^West China Second University Hospital, Sichuan University, Chengdu, China; ^2^West China School of Nursing, Sichuan University, Chengdu, China; ^3^West China Hospital, Sichuan University, Chengdu, China; ^4^Department of Nursing, West China Hospital, Sichuan University, Chengdu, China; ^5^Mental Health Center, West China Hospital, Sichuan University, Chengdu, China

**Keywords:** home care, hospital-based, home care service, willingness, attitude, awareness

## Abstract

**Introduction:**

Hospital-at-home (HaH) services have become increasingly popular. However, the experience of HaH implementation in Asia is inadequate. Therefore, the purpose of this study was to investigate individuals' willingness to accept HaH services and the potential related factors.

**Methods:**

The researchers visited households to select appropriate participants. An online questionnaire survey was conducted among the inhabitants of selected communities. An individual's awareness, willingness to accept HaH services, and demands such as ideal service providers and more detailed information to accept HaH care were investigated. The outcome measure was the willingness to accept HaH services. Chi-square tests and logistic regression models were used to analyze the factors.

**Results:**

A total of 622 subjects participated in this study. The findings indicate that 55.9% of the participants were not aware of HaH services, while most of the subjects (88.4%) were willing to accept them. Regression models indicated that having health insurance (OR = 2.170, 95% CI: 1.003–4.697), an awareness of the necessity of HaH services (OR = 4.721, 95% CI: 2.471–9.019), very much hoping staff from central hospitals would be service providers (OR = 20.299, 95% CI: 5.718–72.068), and somewhat hoping that staff from central hospitals would be service providers (OR = 9.139, 95% CI: 2.714–30.775) were the factors associated with a greater willingness to accept HaH services.

**Conclusion:**

The study indicates that compared to the awareness of HaH care, residents had a greater willingness to accept such care. The willingness to utilize HaH services among individuals was associated with enabling factors, predisposing factors, and HaH-related demand factors.

## Introduction

With increasing health care demand and shortage of hospital beds around the world, the health care system is currently facing the challenge of decreased quality of care (1). The high incidence rate of chronic diseases such as diabetes, cancer, and hypertension (2) has caused many hospitals to be unable to provide sufficient care to satisfy their clients' needs. Moreover, the increasing number of elderly individuals as the population ages contributes to the challenges facing current health systems to satisfy consumers' demands (3, 4). As the proportion of chronic diseases has increased with population aging, the availability of hospital beds has decreased. Even more noteworthy, since the outbreak of the COVID-19 epidemic, according to the World Health Statistics 2021 launch by the World Health Organization (WHO), the number of confirmed cases of COVID-19 has topped 0.153 billion, which has exacerbated the conflict between the supply and demand for medical resources and made it more difficult for people to obtain medical services^1^.

To overcome these complex challenges, an innovative solution that can offer an alternative model of care is needed. With hospital-at-home (HaH) services, originated in 1958, health care professionals provide hospital-level care in patients' homes (1). Gogan suggested that receiving health care at home could help patients recover more rapidly (5). In recent years, HaH care has become increasingly popular as a substitute for inpatient care, and many diseases have been treated through HaH services, including chronic obstructive pulmonary disease and chronic kidney disease (5–10). Compared to inpatient care, HaH services represent substantial cost savings, alleviate the increasing pressures on the inpatient bed supply, and reduce the risks associated with prolonged inpatient care, such as delirium and hospital-acquired infections (11). Mortality and readmission rates do not differ significantly between HaH and inpatient care (6, 7).

The Chinese government has also paid close attention to the implementation of HaH services. The Interim Regulations on HaH Management in Sichuan Province were launched in 2019 and defined the mission, target diseases, leadership, methods, and regulations for HaH services (12). However, compared to several Western countries, such as the United States, United Kingdom, Australia, and Canada, HaH services in China are developing slowly (13). For example, in 2012 a total of 12,200 home health agencies provided home hospital beds in the United States. Insurance coverage for this service was available to 3.43 million patients. A total of 98.6% of these facilities were authorized by Medicare (federal health insurance) and 77.5% were authorized by Medicaid (medical assistance). Home care services were subsidized by Medicare for almost $1.79 billion in 2015 (14, 15). In Canada, health care is mainly provided by a family physician primary care system. Family physicians accompanied by their teams handle more than 80% of disease treatments, and refer patients to appropriate specialists through a referral mechanism when necessary. The Canadian model significantly improves the utilization of medical resources for hospital specialties (16). Due to different cultures, financial development, and compensatory policies, attitudes toward accepting HaH care vary among countries. Despite the prevalence of HaH services in Western countries, studies that explore individuals' willingness to accept HaH services in Asia are limited, and the public perception of HaH services has yet to be well established (13). A deeper comprehension of individuals' attitudes toward HaH care is essential before implementing it in any new setting (17). Moreover, more detailed issues, such as ideal service providers, the proportion of compensation, and the frequency of ward rounds are yet to be determined, and taking individual demands into consideration is crucial in the promotion of HaH development (18).

Therefore, this study adopted the Andersen–Newman behavioral model to explore the willingness of residents to accept HaH services and the potential factors of agreement to HaH services in Asian countries such as China (18), which will be helpful for obtaining a deeper understanding of residents' opinions concerning HaH services and in providing reliable evidence for HaH promotion and service improvement to ensure that targeted services can be offered.

## Methods

### Study Design

This was a cross-sectional study carried out with 622 residents of Chengdu City by using a personal characteristics questionnaire and a questionnaire that assessed respondents' awareness of and willingness to accept HaH services.

### Participants

The initial sample size (*n* = 600) was calculated by the formula *N* = *Z*^2^× {*P*× (1−*P*)}÷*E*^2^(Z = 1.96, *E* = 4%, and *P* = 0.5). Participants were selected through convenience sampling. Because of the possibility of survey dropout, 700 participants were invited to finish the survey. Volunteers who met the following criteria were enrolled in this study: (1) >18 years old; (2) no cognitive impairment or psychiatric disorder; and (3) permanent residents of Chengdu City. Of the 700 residents invited to participate, 650 answered the questionnaire, and of these, 622 (88.9%) provided complete and usable data to be included in the data analysis. Only complete data were analyzed.

### Sample Collection

To ensure that the process of data collection would go smoothly, the researchers discussed with several community leaders in Chengdu. With the consent of the people in charge, the surveys were administered to subjects from five communities in Chengdu City. Through lectures, the researchers explained the HaH concept and function, as well as the proposal and precautions of the survey. After the lectures were completed, three researchers visited households in the community to select subjects who met the inclusion criteria. After the link to the online questionnaire was sent to the subjects by a WeChat QR code, the subjects completed the questionnaires by themselves. During this process, the researchers always answered the subjects' questions to ensure that the subjects fully understood each item of the questionnaire to improve the accuracy of each answer. Through an online questionnaire, the processes of data collection and entry were integrated. The software package WJX (www.wjx.com) was used to collect data. WJX is a professional online platform widely used in several fields that includes data collection, custom reports, and survey result analysis. More than 0.1 billion individuals in China have registered with WJX software. To ensure rigor in the sample collection process and reduce bias, strict quality control measures were adopted. Misfillinging or omission of filling the questions was avoided by preset single-choice, multiple-choice, and fill-in-the-blank questions. The questionnaires could not be submitted unless they were completed. One person could only fill in the questionnaire once after all the topics were completed. Each questionnaire was screened by automatic screening rules and manually checked by the researchers after submission. Any answer that did not meet the requirements, such as only one option was selected or the questionnaire was finished within 60 s, was marked as invalid and removed.

### Instruments

#### Personal Characteristics Questionnaire

A personal characteristics questionnaire was designed to collect data on the participants' general characteristics, including their gender, age, educational background, occupation, income, family income, and health status, such as whether they currently or previously had any chronic disease, type of chronic disease, family's health status, medication usage, family's medication usage, and family's visual acuity status.

#### Questionnaire on Residents' Awareness of, Willingness to Accept, and Demands Regarding HaH Services

The researchers designed a questionnaire consisting of 12 multiple-choice items to investigate Chengdu residents' willingness to accept HaH services and potential related factors. This questionnaire was constructed after reading related literature and was revised by many experts; Cronbach's α coefficient (0.821) and Kaiser–Meyer–Olkin measure (0.948) were calculated as reliability and validity indicators. The variables were as follows: whether the subject had heard about HaH services; whether the subject had accepted any HaH services; the necessity of launching HaH services; the reasons why the subject believed that HaH services were important; an awareness of the extent of HaH service reimbursement; the ideal service provider; the frequency of ward rounds by doctors; the frequency of nursing ward rounds; the ideal expense account for family income; the attitude toward doctors and nurses from the central hospitals that provide the services; and willingness to accept HaH services. The primary dependent variable was the willingness of residents to accept HaH services.

### Independent and Dependent Variables

The Andersen–Newman behavioral model was constructed by Professor Ronald in 1968 and is one of the classic models for studying the utilization of health services. The model involves three types of determining factors that affect an individual's utilization, namely, predisposing, enabling, and need factors. Predisposing factors refer to personal sociocultural position prior to health service utilization, which consist of demographic characteristics, social structure, and health beliefs. Demographic characteristics indicate one's basic condition, such as age and gender. Social structure includes one's educational background, occupation, social network, and perception of the health service system (18). Therefore, we inquired about individuals' age, gender, educational background, occupation, whether they had heard about HaH care, whether they had accepted HaH services, the necessity of launching HaH services, and their awareness of the extent of HaH service reimbursement as predisposing factors. Enabling factors include the capacity to obtain health services and the availability of health services, such as income and health insurance (18). Therefore, an individual's monthly income, family monthly income, and health insurance status were defined as enabling factors. Need factors indicate the cognitive requirements of individuals for health services, including their health status and disease conditions (18). As for service demands, service provider requirements were also taken into consideration and were defined as the HaH-related demand factor. The dependent variable of the study was willingness to accept HaH services.

### Statistical Analysis

The data were analyzed with the SPSS 21.0 software package. Frequency and percentage statistics were used to analyze the participants' predisposing, enabling, need, and HaH-related factors. The results of the questionnaire on the participants' awareness of, willingness to accept, and demands regarding HaH are also presented as frequencies and percentages. The associations among the independent variables and dependent variable were evaluated by using the chi-square test, with *P* < 0.05 considered to be statistically significant. Before a logistic regression was performed, the correlations among the independent variables were also analyzed. To bypass the impact of collinearity between the independent variables on the final findings, stepwise (forward) logistic regression models were run to minimize the effect of the correlation between variables, including only the independent variables associated with the dependent variable.

### Ethics

The study was approved by the Ethics Committee of West China Hospital, Sichuan University (No. 848). All participants were informed of the details of this study and agreed to participate in the survey. Written informed consents were provided by each participants.

## Results

### Participant Characteristics

A total of 622 residents of Chengdu City participated in this study (including 166 males and 456 females). Most were between 30 and 50 years old (mean ± SD: 40.22 ± 12.95). Of the respondents, 36.35% had a bachelor's degree or above, 47.9% were managers, public servants, or professional personnel, and 90.0% had health insurance. A total of 17.4% currently or previously had a chronic disease, while 46.5% of participants' relatives currently or previously had a chronic disease.

### Residents' Awareness of, Willingness to Accept, and Demands Regarding HaH Services

The data from this study indicate that 55.9% of participants were not aware of HaH services, while most of the subjects (88.4%) were willing to accept them. Only 2.4% of residents had accepted HaH care before this survey. In this study, 418 (67.2%) of individuals thought that HaH services were necessary. A total of 360 (57.9%) residents were willing to accept better companionship, and 253 (40.7%) were willing to accept this for convenience. Details are shown in Table 1.

**Table 1:** Participant characteristics, awareness of, willingness to accept, and demands regarding HaH services.

**Variables**	**Total (*n* = 622)**
**Gender**	
Female	166 (26.7%)
Male	456 (73.3%)
**Age**	
18–30	153 (24.6%)
31–50	362 (58.2%)
51–60	83 (13.3%)
>60	24 (3.9)
**Educational background**	
Primary school and below	53 (8.5%)
Junior high school	104 (16.7%)
High school	49 (7.9%)
College degree	190 (30.5%)
Bachelor degree and above	226 (36.3%)
**Occupation**	
Mangers, public institutions, or professional personnel	298 (47.9%)
Workers (production or transportation equipment operators and related personnel)	31 (5.0%)
Farmers	58 (9.3%)
Retirees	116 (18.6%)
Others	21 (3.4%)
Unemployed	98 (15.8%)
**Health insurance**	
Yes	560 (90.0%)
No	62 (10.0%)
**Marital status**	
Spinsterhood	141 (22.7%)
Married	468 (75.2%)
Others	13 (2.1%)
**Number of offspring**	
None	155 (24.9%)
1	304 (48.9%)
2	145 (23.3%)
3 and more	18 (2.9%)
**Family monthly income**	
6,000 below	188 (30.2%)
6,000–8,000	101 (16.2%)
8,001–−10,000	82 (13.2%)
10,001–15,000	93 (15.0%)
15,001–20,000	80 (12.9%)
20,000 and more	78 (12.5%)
**Whether currently or previously had any chronic disease**	
Yes	108 (17.4%)
No	514 (82.6)
**Disease condition**	
High blood pressure	40 (6.4%)
Coronary heart disease	6 (1.0%)
Diabetes	19 (3.1%)
Cancer	23 (3.7%)
Other	18 (2.9%)
**Family suffering from chronic disease**	
Yes	289,46.5%
No	333,53.5%
**Family disease condition**	
High blood pressure	211 (33.9%)
Coronary heart disease	58 (9.3%)
Diabetes	127 (20.4%)
Cancer	30 (4.8%)
Other	11 (1.8%)
**Awareness**	
Yes	274 (44.1%)
No	348 (55.9%)
**Accepted before**	
Yes	15 (2.4%)
No	607 (97.6%)
**Necessity**	
Yes	418 (67.2%)
No	204 (32.8%)
**Awareness of expense of HaH services reimbursement**	
Available to reimburse	153 (24.6%)
Not available to reimburse	78 (12.5%)
Unclear	391 (62.9%)
**Ideal service provider**	
Doctors and nurses of community hospitals	141 (22.7%)
Doctors and nurses of central hospitals	373 (60.0%)
Do not mind	97 (15.6%)
Other	11 (1.8%)
**Frequency of ward rounds by doctors**	
Once a week	95 (15.3%)
Twice or three times a week	173 (27.8%)
4–5 times a week	31 (5.0%)
Once a day	67 (10.8%)
Twice a day	74 (11.9%)
According to my demands	182 (29.3%)
**Ideal proportion of HaH expenses that account for family income**	
20% below	424 (68.2%)
20−30%	145 (23.3%)
30−40%	31 (5.0%)
40% and more	22 (3.5%)
**The attitude toward staff from central hospitals providing service**	
Very much hoping	338 (54.3%)
Somewhat hoping	226 (36.3%)
Do not mind	40 (6.4%)
Do not hope	18 (2.9%)
**Willingness**	
Yes	550 (88.4%)
No	72 (11.6%)

### Chi-Test of the Willingness to Accept HaH Services by Predisposing Variables, Enabling Variables, Need Factors, and HaH-Related Demands

The chi-square test detected the potential factors that affected the likelihood of agreeing to HaH services. The participants' educational background, occupation, health insurance status, source of revenue, monthly income, family monthly income, awareness of HaH services, ideal frequency of ward rounds, ideal service providers, and attitudes toward doctors and nurses from the central hospitals that provided services may affect their willingness to accept HaH care (Table 2).

**Table 2:** Chi-test of the willingness to accept HaH services.

	**Resident's personal characteristics**	**Categories**	**Willingness to accept HaH services**		***P-*Value**
			**Yes**	**No**	
			***n* (%)**	***n* (%)**	
Predisposing	Educational background	Primary school and below	45 (8.2)	8 (11.1)	<0.001
factors		Junior high school	81 (14.7)	23 (31.9)	
		High school	39 (7.1)	10 (13.9)	
		College degree	172 (31.3)	18 (25.0)	
		Bachelor degree and above	213 (38.7)	13 (18.1)	
	Occupation	Mangers, public institutions, or professional personnel	280 (50.9)	18 (25.0)	<0.001
		Workers (production or transportation equipment operators and related personnel)	23 (4.2)	8 (11.1)	
		Farmers	50 (9.1)	8 (11.1)	
		Retirees	101 (18.4)	15 (20.8)	
		Others	15 (2.7)	6 (8.3)	
		Unemployed	81 (14.7)	17 (23.6)	
	Awareness	Yes	259 (47.1)	15 (20.8)	<0.001
		No	291 (52.9)	57 (79.2)	
	Necessity	Yes	402 (73.1)	16 (22.2)	<0.001
		No	148 (26.9)	56 (77.8)	
Enabling factors	Health insurance	Yes	507 (92.2)	53 (73.6)	<0.001
		No	43 (7.8)	19 (26.6)	
	Family monthly income	6,000 below	152 (27.6)	36 (50.0)	0.001
		6,000–8,000	87 (15.8)	14 (19.4)	
		8,001–10,000	75 (13.6)	7 (9.7)	
		10,001–15,000	86 (15.6)	7 (9.7)	
		15,001–20,000	78 (14.2)	2 (2.8)	
		20,000 and more	72 (13.1)	6 (8.3)	
HaH-related	Ideal service provider	Doctors and nurses of community hospitals	128 (23.3)	13 (18.1)	<0.001
demand factors^a, b^		Doctors and nurses of central hospitals	344 (62.5)	29 (40.3)	
		Do not mind	68 (12.4)	29 (40.3)	
		Other	10 (1.8)	1 (1.4)	
	Ideal frequency of ward rounds by doctors	Once a week	87 (15.8)	8 (11.1)	0.002
		Twice or three times a week	161 (29.3)	12 (16.7)	
		4–5 times a week	30 (5.5)	1 (1.4)	
		Once a day	57 (10.4)	10 (13.9)	
		Twice a day	56 (10.2)	18 (25.0)	
		According to my demands	159 (28.9)	23 (31.9)	
	Attitude toward staffs from central hospitals provide service	Very much hoping	324 (58.9)	14 (19.4)	<0.001
		Somewhat hoping	200 (36.4)	26 (36.1)	
		Do not mind	21 (3.8)	19 (26.4)	
		Do not hope	5 (0.9)	13 (18.1)	

^a^*HaH, Hospital-at-Home services*.

*^b^Missing data are not included in the values*.

### Correlation Analysis Among the Independent Variables

The results of the correlation analysis tested the collinearity between the independent variables. The results indicated that there are significant correlations between the variables, especially between demographic variables such as educational background, occupation, and health insurance, which may affect the research results. Therefore, rigorous statistical methods were required. Stepwise (forward) logistic regression models were run to minimize the effect of the correlation between variables (Table 3).

**Table 3:** Correlation between the independent variables.

	**Educational background**	**Occupation**	**Health insurance**	**Family monthly income**	**Awareness**	**Necessity**	**Ideal service providers**	**Ideal frequency of ward rounds**	**The attitude toward staff from central hospitals providing service**
Educational background	1.000								
Occupation	−0.604**	1.000							
Health insurance	0.378**	−0.276**	1.000						
Family monthly income	0.600**	−0.512**	0.237**	1.000					
Awareness	−0.292**	0.307**	−0.187**	−0.296**	1.000				
Necessity	−0.349**	0.338**	−0.225**	−0.316**	0.351**	1.000			
Ideal service providers	−0.174**	0.137**	−0.073	−0.117**	0.127**	0.136**	1.000		
Ideal frequency of ward rounds	−0.236**	0.152**	−0.042	−0.112**	0.160**	0.124**	0.227**	1.000	
The attitude toward staff from central	−0.173**	0.174**	−0.126**	−0.239**	0.135**	0.300**	0.114**	0.114**	1.000
hospitals providing service					

**P < 0.001.

### Comparison of the Willingness to Accept HaH Services by Predisposing Variables, Enabling Variables, Need Factors, and HaH-Related Demands

A stepwise logistic regression model was used to identify the variables associated with the willingness to accept HaH services, and the final regression model indicated that being enrolled in health insurance (OR = 2.170, 95% CI: 1.003–4.697), an awareness of the necessity of HaH services (OR = 4.721, 95% CI: 2.471–9.019), very much hoping that staff from central hospitals would be the service providers (OR = 20.299, 95% CI: 5.718–72.068), and somewhat hoping that staff from central hospitals would be the service providers (OR = 9.139, 95% CI: 2.714–30.775) were the factors associated with a greater willingness to accept HaH services (Table 4).

**Table 4:** Comparison of the willingness to accept HaH services by predisposing variables, enabling variables, need factors, and HaH-related demands.

**Independent variables**	***B***	**SE**	**Wals**	**df**	***P-*Value**	**OR**	**95% confidence interval**	
							**Lower**	**Upper**
Health insurance (REF: no)	0.775	0.394	3.867	1	0.049	2.170	1.003	4.697
Necessity (REF: no)	1.552	0.330	22.083	1	0.000	4.721	2.471	9.019
Ideal service provider (REF: doctors and nurses of community hospitals)			10.703	3	0.013			
Doctors and nurses of central hospitals	0.163	0.402	0.164	1	0.686	1.177	0.535	2.587
Do not mind	−0.962	0.433	4.938	1	0.026	0.382	0.163	0.893
Other	−0.048	1.177	0.002	1	0.967	0.953	0.095	9.562
Attitude toward staff from central hospitals providing service (REF: do not hope)			36.766	3	0.000			
Very much hoping	3.011	0.646	21.688	1	0.000	20.299	5.718	72.068
Somewhat hoping	2.213	0.619	12.756	1	0.000	9.139	2.714	30.775
Do not mind	0.755	0.684	1.218	1	0.270	2.127	0.557	8.126
Constant	−2.189	0.880	6.182	1	0.013	0.112		

## Discussion

To our knowledge, this study is the first to explore individuals' awareness of and willingness to accept HaH services that focuses on the demands related to HaH services based on the Andersen–Newman behavior model. The findings might serve as guidelines for future HaH promotion and implementation. Our findings emphasize the impact of HaH-related variables such as the awareness of HaH services, service demands, and reimbursement demands on the acceptance of HaH services among individuals in Chengdu City. In contrast to a similar study conducted in Singapore, the vast number of subjects in this study would accept HaH services (13). This phenomenon can be explained by the differing health care service models and resource allocation between China and Singapore. Singapore not only has a dual system of health care services, with a public system provided by the government and a private system provided by the private sector but also has 24.60 medical doctors and 62.43 nursing personnel per 10,000 people (19). In China, medical and health institutions are divided into public and socially run categories. Public medical institutions are the main medical service providers, and they are supplemented by socially run medical institutions, with 22.27 medical doctors and 30.83 nursing personnel per 10,000 people, which are both lower than in Singapore (19). Therefore, we infer that Chinese residents may have to spend more time waiting for effective treatment, as China's health care human resources are more limited. HaH services have been proven to be effective in saving time and alleviating the shortage of medical resources; therefore, Chinese residents are more willing to accept them. Individuals' willingness to accept HaH services is connected to their health insurance status, awareness of their necessity, and attitudes toward staff from the central hospitals that offer HaH services.

The findings of this study indicate that compared to the awareness of HaH services, there was a greater willingness to accept HaH care among the subjects. Because of the shortage of medical resources in recent years, healthcare professionals cannot provide adequate medical services to every patient, which results in patients' needs not being fully met and leads to poor healthcare experiences (20, 21). In addition, the unfamiliarity of the hospital environment can harm patients' mental health, which is not conducive to their recovery (22). Prolonged hospitalization can lead to additional risks, such as delirium and infections (23–28). These problems are mitigated by HaH services, which might be why most of the subjects were willing to accept them. However, there is inadequate advertising and utilization of HaH services. Therefore, the awareness of HaH care is not high. Accordingly, concrete measures are needed to raise awareness of the concept, strengths, and functions of HaH services, such as information disseminated through television, newspapers, and applications, to increase residents' understanding of these services. Individuals' awareness of the necessity of HaH services is related to their willingness to accept HaH services. According to the Andersen–Newman behavioral model, an individual's perception of a medical service before receiving it can influence his or her willingness to accept this service (18). After hearing an explanation of the concept and function of HaH services, subjects might be more aware of the necessity of developing HaH care to ameliorate the shortage of medical resources. Therefore, those who are more aware of the necessity of HaH services might perceive more benefits and exhibit a higher willingness to accept HaH services.

As an enabling factor, health insurance coverage is associated with the willingness to accept HaH services. This result is similar to a study that argued that individuals enrolled in health insurance are more willing to maintain contact with family doctors (29). A previous study confirmed that health insurance status varies by socioeconomic status. Individuals not enrolled in health insurance may have a lower socioeconomic status; therefore, they cannot afford the full cost of HaH care (30, 31). Being covered by health insurance can reduce the cost of medical services and alleviate patients' stress, which enables them to be available to accept health services (29). Accordingly, individuals not enrolled in health insurance cannot afford the fees for HaH services. Therefore, wider enrollment in health insurance is key to the promotion of HaH services. More mature compensatory policies are needed, and the experience of developed countries is enlightening. For instance, in Norway, home care is free for patients, and in Canada, it is funded by the government (32). Moreover, the results of this study showed that 391 participants were not clear about the cost of HaH services, and most hoped that it would be <20% of their monthly family income. Many countries where HaH services have been widely implemented, such as Australia and the UK, have single-payer systems and strong imperatives to keep medical costs low; related systems need to control the overall cost of HaH care and set proper charging standards (33).

The vast number of the subjects in this study (54.3%) hoped that staff from central hospitals would offer HaH services. This study also found that the participants who very much or somewhat hoped that staff from central hospitals would be service providers were inclined to be more accepting of HaH services. However, in China, HaH care is mostly performed by community staff with only a college degree or below and limited medical skills to meet client requirements (34, 35). Moreover, highly qualified health staff in China always work in central hospitals and do not have time to provide HaH services (35). This phenomenon can be explained by the siphon effect of high-grade hospitals in China; these hospitals attract both large numbers of patients, which gives them a strong “resource siphon” ability, and qualified doctors to actively practice there for more opportunities and career resources (36). However, the workload in central hospitals in China is too heavy for these staff to provide extra HaH services (35). In Spain, each HaH unit consists of at least an internal medicine specialist and a general physician who incorporate specialists such as pulmonologists, geriatricians, and general surgeons. Nurses who provide HaH services must also be trained in hospital specialties and obtain a qualification certificate (37). In Sweden, nurses who provide home care services are required to have at least a bachelor's degree, and a master's degree is preferable (38). Therefore, related departments should make efforts to encourage health systems to develop HaH services and provide adequate resources for this development to inspire well-trained professionals to participate (13). The experiences of other countries can be studied to understand how to launch HaH services. For instance, it would be useful to develop a curriculum on medical care at home, promote practical home care skills, and train students on the use of portable medical devices to cultivate talent with at least a bachelor's degree in home care (35).

Interestingly, one of the findings of this research contradicts previous literature: socioeconomic factors were not associated with an individual's willingness to accept HaH care (30, 31). As there is a linkage between enrollment in health insurance and an individual's educational background, occupation, and income, it appears that enrollment in health insurance might act as a mediating variable between socioeconomic factors and willingness to accept HaH services. People who have high earnings with higher educational backgrounds care a great deal about their health; therefore, they pay health insurance enrollment fees (39). However, further analysis is needed to better explain this phenomenon. Individuals' health status was also not associated with their willingness to accept HaH services, which might be explained by the fact that there were only 622 participants in this study, and most did not have a chronic disease. Further investigation is also needed on this issue.

China has a total of 1.4 billion people, which accounts for nearly one-third of the total population of Asia, and there are similarities between the health care delivery systems in mainland China and both Japan and South Korea. Therefore, because of the similar health care systems and population ratios in China, we believe that the results of this study may have some generalizable implications. However, due to the differences in the per capita medical human resources and economic development levels, whether the results of this study are representative of Asia requires further discussion.

This study has certain limitations. First, it used a convenience sampling method, not random sampling, which may have resulted in bias. Second, the sample size was only 622, which may lead to the sample being unrepresentative. For example, the gender ratio in Chengdu is nearly 1:1 (50.54%:49.46%) (40); however, male respondents accounted for only 26.7% of the sample in this study. A smaller sample size might fail to reflect the real willingness of the male population. In addition to the male population, the other groups that accounted for a minor proportion, such as workers' estimated willingness (e.g., production or transportation equipment operators and related personnel), may be affected. Third, this study was a cross-sectional study that was unable to explore how individuals' willingness to accept HaH care is shaped. Therefore, longitudinal studies are needed. Fourth, the survey utilized an online method to collect data, and those who were not familiar with the internet may be under-represented. Thus, this study's participants might not represent elderly individuals with more healthcare needs, whose views are also important. However, this study investigated the willingness of residents to accept HaH services and demonstrated the gap between residents' actual demands and willingness to accept HaH services. The results of this study are significant for both the medical system and residents. In terms of the medical systems' viewpoints, a better understanding of residents' perceptions and demands can help develop more targeted services and effective advertising policies to lead to a higher willingness to accept HaH services. Therefore, HaH services can be widely accepted to alleviate medical resource insufficiency. In terms of residents' viewpoints, a better understanding of their opinions and demands can result in better service, more reasonable charging standards, and regular work procedures of HaH care to ensure that people can truly benefit from HaH services.

## Conclusion

This study shows that there was a lower awareness of but higher willingness to accept HaH services among the participants, which indicates that the awareness of HaH services among the residents of Chengdu City has room for growth. The willingness to accept HaH services among individuals was associated with enabling factors such as health insurance enrollment, predisposing factors such as an awareness of the necessity of HaH services, and HaH-related demand factors such as attitudes toward staff from central hospitals as service providers. Therefore, effective policies and practical measures must be created to motivate the development of HaH services.

## Data Availability Statement

The original contributions presented in the study are included in the article/Supplementary Material, further inquiries can be directed to the corresponding author/s.

## Ethics Statement

The studies involving human participants were reviewed and approved by the Ethics Committee of West China Hospital, Sichuan University. The patients/participants provided their written informed consent to participate in this study. Written informed consent was obtained from the individual(s) for the publication of any potentially identifiable images or data included in this article.

## Author Contributions

JW and YW were involved in the design of the study, acquisition of data, and development of the statistical framework, and they reviewed the manuscript. MW and XH were involved in the study design and development of the analysis framework. SX and HH developed the statistical framework for data analysis, conducted the statistical analysis, interpreted the data, and drafted the manuscript. All authors read and approved the final manuscript.

## Funding

This research was supported by the Science and Technology Department of Sichuan Province Fund Project (Program Nos: 2021YFS0151 and 2021JDR0188) and the West China School of Nursing, Sichuan University (HXHL19012).

## Conflict of Interest

The authors declare that the research was conducted in the absence of any commercial or financial relationships that could be construed as a potential conflict of interest.

## Publisher's Note

All claims expressed in this article are solely those of the authors and do not necessarily represent those of their affiliated organizations, or those of the publisher, the editors and the reviewers. Any product that may be evaluated in this article, or claim that may be made by its manufacturer, is not guaranteed or endorsed by the publisher.

## Footnotes

^1^ https://www.who.int/emergencies/diseases/novel-coronavirus-2019#
